# Medical graduate views on statistical learning needs for clinical practice: a comprehensive survey

**DOI:** 10.1186/s12909-019-1842-1

**Published:** 2019-12-31

**Authors:** Margaret MacDougall, Helen S. Cameron, Simon R. J. Maxwell

**Affiliations:** 1 Centre for Population Health Sciences, Usher Institute, Edinburgh Medical School, University of Edinburgh, Teviot Place, Edinburgh, EH8 9AG UK; 2 Aston Medical School, Aston University, Birmingham, B4 7ET UK; 3 Internal Medicine Office, Medical Education Centre, University of Edinburgh, Western General Hospital, Edinburgh, EH4 2XU UK

**Keywords:** Clinical practice, Critical appraisal, Curriculum design, Statistical learning, Statistics education research, Undergraduate medicine

## Abstract

**Background:**

This paper seeks to contribute to a reputable evidence base for required competencies across different topics in statistics and probability (statistical topics) in preparing medical graduates for clinical practice. This is in order to inform the prioritization of statistical topics within future undergraduate medical curricula, while exploring the need for preparing tomorrow’s doctors to be producers, and not merely consumers, of statistics.

**Methods:**

We conducted a comprehensive online survey from July 2013 to August 2014 for a target group of 462 medical graduates with current or prior experience of teaching undergraduate medical students of the University of Edinburgh of whom 278 (60.2%) responded. Statistical topics were ranked by proportion of respondents who identified the practice of statistics, *performing statistical procedures or calculations using appropriate data*, as a required competency for medical schools to provide in preparing undergraduate medical students for clinical practice. Mixed effects analyses were used to identify potential predictors for selection of the above competency and to compare the likelihood of this selection for a range of statistical topics versus critical appraisal.

**Results:**

Evidence was gleaned from medical graduates’ experiences of clinical practice for the need for, not only a theoretical understanding of statistics and probability but also, the ability to practice statistics. Nature of employment and statistical topic were highly significant predictors of choice of the practice of statistics as a required competency ((F = 3.777, p < 0.0005) and (F = 45.834, p < 0.0005), respectively). The most popular topic for this competency was graphical presentation of data (84.3% of respondents) in contrast to cross-over trials for the competency understanding the theory only (70.5% of respondents). Several topics were found to be more popular than critical appraisal for competency in the practice of statistics.

**Conclusions:**

The model of medical graduates as mere consumers of statistics is oversimplified. Contrary to what has been suggested elsewhere, statistical learning opportunities in undergraduate medicine should not be restricted to development of critical appraisal skills. Indeed, our findings support development of learning opportunities for undergraduate medical students as producers of statistics across a wide range of statistical topics.

## Background

The potential impact on clinical practice of a collaborative approach between statisticians and medical graduates in improving the quality of learning in statistics within the undergraduate medical curriculum is evident from existing literature. For example, findings dating back to the 1980s report that practicing physicians struggle to interpret, or evaluate the interpretation of, clinical findings in medical literature because they lack a working knowledge of statistics [1].

The latter finding is particularly troublesome given the recognized demand on practicing physicians “to demonstrate that they can reach correct diagnoses using both clinical and statistical data” [2]. Furthermore, Horton and Switzer provide evidence for a continued increasing trend, previously reported in 1992, in level of complexity of statistical methods used to report clinical research findings in the New England Journal of Medicine (NEJM). Indeed, they note that this includes use of methods “not typically included in introductory or second-level statistics courses”. In turn, they express the concern that difficulty in comprehending statistical content in such cases may hinder disemination of study findings among clinicians [3]. In seeking to carry over statistical techniques to the analysis of *their own patient data*, clinicians who lack the prequisite training to test model assumptions may generate misleading results, while duped by the ease of use of the available software.

The legitimacy of this concern has been confirmed by Altman and Bland through their reflections on the statistical errors in the medical literature, where they also note that “Statistics is much more subjective (and difficult) than is usually acknowledged (this is why statisticians have not been replaced by computers).” and identify the long-standing problem of “frequent misuse of medical statistics” as being a concern to statisticans [4]. The latter problem, which has been identified in high-ranking clinical journals [5], among others [6], is compounded by an “increasing pressure” on “physcicians to make use of techniques that they do not fully understand” [4]. As Altman and Bland note, this increasing pressure, which is particularly evident at the early stages of clinical careers in general, is influenced by the requirement to publish for career advancement and prestige. It is also influenced by a lack of funding resources to support input from a statistician, as is frequently the case for specialist registrars, and more generally, from the fact that demand for medical statisticians exceeds supply. Medical graduates in this position cannot reasonably be expected to identify misleadingly analysed data unless they have been trained in assumptions testing using their own statistical calculations and analyses. The alternative is that through overreliance on published clinical findings, physcians may arrive at “wrong diagnostic or therapeutic decisions and so put patient health or even lives at risk” [4].

The plausability of this outcome is illustrated by Gigerenzer et al. through reference to the challenge that physicians face in translating conditional probabilites from diagnostic statistics into a meaningful prediction about disease status when a patient presents with a positve diagnostic test result. Here, experiential learning through practical application of Bayes’ Theorem is a sensible approach to preparing medical graduates for patient-doctor interactions and managing the translation of published results, including sensitivity and other conditional probabilities into a valid prognosis for the patient. Relatedly, there are ethical issues at stake where physicians are not empowered to communicate relevant statistical findings and associated levels of uncertainty arising from probabilistic reasoning to their patients. Specifically, the “goals of informed consent and shared decision making” [7] are undermined. Albeit inadvertently, the integrity of the patient-doctor relationship is also undermined, as the doctor is compelled to deliver an “illusion of certainty” to meet the expectations of the patient.

The problem of statistical literacy in this sense can in turn contribute to the recognized phenomenon of variation in recommended treatment regimens for identical conditions across different physician specialties, medical practices and geographical regions within the same country, suggesting that “local habits” take precedence over common appreciation of best evidence [7]. Medical educators can make some headway in addressing these critical issues by providing better opportunities for undergraduate medical students to acquire competencies in both the theory and practice of statistics.

Clearly then, there is a call to entertain the clinician’s voice *as informed by their own working practices* in defining statistical learning needs for tomorrow’s doctors. Despite the above observations from the literature, we addressed this call with an openness to the possibility that medical graduates believe that very little learning in statistics is required for clinical practice.

Some insight into the utility of learning statistics and probability in medicine was reflected in a 2007 survey-based study [8, 9]. This particular study involved responses from 130 (27.5%) out of a population of approximately 473 clinicians affiliated with the University of East Anglia. The above study is helpful in highlighting that for a majority of broad “work activities”, a high proportion of those respondents who performed the activity in their existing job roles deemed an understanding of statistics and probability to be useful for that activity. This proportion was approximately 90% for each of the activities “accessing clinical guidelines and evidence summaries, explaining levels of risk to patients, assessing medical marketing and advertising material, interpreting the results of a screening test, reading research publications for general professional interest and using research publications to explore non-standard treatment and management options.” Nevertheless, the statistical queries raised via the 2007 survey were of a relatively general nature, without a focus on topic-specific content needs for undergraduate medical curricula.

The principal aims of our study were therefore:

- to employ a structured survey to obtain a comprehensive evidence base across a range of clinical specialities defining which topics in statistics and probability (henceforth “statistical topics”) physicians have deemed to be useful *within their own clinical practice*;
  and
- to use the above evidence base to present a profile for the relative importance of individual statistical topics in terms of the competencies *carry out the procedure or calculate the statistic(s) using appropriate data* (*engage in the practice of statistics*) and *understand the theory only*.

The secondary aim was to employ mixed effects analyses to identify potential predictors for respondents opting for competency in the practice of statistics and to compare the likelihood of this choice for a range of statistical topics with that of critical appraisal.

We identified these aims to inform the prioritization of statistical topics within future undergraduate medical curricula, while exploring the need for preparing tomorrow’s doctors to be producers, and not merely consumers, of statistics.

## Methods

### Establishing a well-defined target population with an accurate response rate

This study is based on an online survey targeting medical graduates who were also identified as current or prior teachers of undergraduate medical students (medical undergraduates) of the University of Edinburgh (UoE). We defined the target group in this way in order to ensure a good estimate for the denominator in determining the response rate, to ensure that critical survey questions pertaining to teaching were appropriate and to allow meaningful comparisons with previous research.

Eligible members of the target group and their corresponding up-to-date email addresses were identified by the Principal Investigator (PI) using existing lists provided by UoE administrators and by extensively revising these lists based on:

- existing content on the UoE Electronic Medical Curriculum;
- email and telephone correspondence with administrative and clinical staff;
- details provided on a range of hospital and university webpages;
  and
- contact details within the National Health Service (NHS) network database of practicing clinicians.

The final target group was identified by the PI through cross-examination of the information from the above sources, noting that source d) was not always reliable. With a view to reducing response bias, the three members from the research team who met the requirements for membership of the target group were excluded from that group. Potential duplicates arising from individuals with multiple email addresses were checked through at least one of a) to d), above.

### Pre-testing and finalizing the survey

The PI designed an online draft questionnaire by means of a secure online survey system. They also carried out multiple test-runs of the survey prior to distribution. This included reviewing of question content and confirmation that both skip logic and prompts to notify respondents that a previously unanswered question required a response were working satisfactorily. Two of the research team who were excluded from the target population provided feedback, each in their capacities as both curriculum leads and clinical professionals. Appropriate changes were then agreed on and implemented by the PI in order to optimize clarity and focus. This approach was enhanced by a formal feedback exercise involving a comprehensive list of evaluation questions completed by five consenting members of the target group.

### Key features of study questionnaire

By means of the study questionnaire, we first provided potential respondents with the invitation (Q. 1) *Please select ALL options which describe the nature of your employment*. together with the options *Clinical practice*, *Academic research*, *Academic teaching* and *Other (please specify)*. Since obtaining topic-specific feedback on the statistical learning needs of medical undergraduates was central to this study, the principal question was situated early on as Q. 2. For ease of reference, we provide the stems of the first (main) part and the second part of this question below.

Stem for first (main) part of Q. 2:

Please use your own experience as a medical graduate to identify those competencies in statistics and probability that medical schools need to provide within the undergraduate medical curriculum to ensure thorough preparedness of their new medical graduates for clinical practice.

*For each of the topics listed below, select the most appropriate response.*

*The option ‘don’t know’ is available for your use wherever appropriate.*

Stem for second part of Q. 2:

*Please also use the corresponding “What’s missing?” box if you feel the list is incomplete, while specifying the corresponding drop-down menu option you would have chosen if the item had been listed.****The accuracy of your responses to this question is critical so please proof-check your responses to check that you haven’t skipped any topics. Thank you for your patience.***

For each of the 52 listed statistical topics which followed, we offered potential respondents a drop-down list comprising the following five options: *understand the theory only*, *carry out the procedure or calculate the statistic(s) using appropriate data*, *both of the above*, *neither*, and *don’t know*. We chose these categories in order to gain a complete overview of medical graduate perspectives on their roles as ‘consumers’ (understanding the theory) and ‘producers’ (carrying out statistical procedures or calculations) of statistics.

The listed topics were derived from the PI’s experience of almost a decade in meeting the statistical learning needs of medical undergraduates engaged in short-term research projects through consultations. These students were predominantly from Year 4 of a five-year medical curriculum, but also included students, who, based on academic merit, had been admitted to the honours year of any one of 20 available biomedical science degree programmes between Years 2 and 3 of their medical degree. Typically, such students would have had exposure to statistics through the following opportunities:

- public health-oriented teaching on study design, critical appraisal, diagnostic statistics and concepts of epidemiology in Year 2, delivered through formal lectures complemented with short case-study assignments;
- depending on choice of honours degree, bespoke learning in statistics through using a statistical package, such as GraphPad Prism, R or SPSS, occasionally involving a short computer-based course covering statistical hypothesis testing, up to the level of Analysis of Variance (ANOVA);
- a single lecture in each of Years 3 and 4 providing:
  - advice on research planning, including data preparation for statistical analysis;
  - topical examples on the need for a) assumptions testing prior to choice and application of statistical procedures and b) avoiding misconceptions through awareness of the phenomenon *regression to the mean*;
    and
  - pointers to online tutorials in statistics covering use of the statistical package SPSS, types of data (as a prelude to hypothesis testing), and fundamentals of: hypothesis testing, questionnaire design, ANOVA and sample size calculations.

In collaboration with learning technologists, the PI had developed a comprehensive knowledgebase and a corresponding electronic search index within their institution’s electronic medical curriculum. The content was largely informed by queries raised in the above consultations [10]. The content of the index informed the initial list of statistical topics to include in the study questionnaire. The wording of items in this list was in turn honed based on feedback concerning appropriate level of detail obtained from medical graduates during pre-testing of the survey.

We invited respondents to identify their status (“Current”, “Previous” or “Never”) as an educator of medical undergraduates of the UoE (Q. 4). We also collected clinical specialties (Q. 12) and invited respondents to identify their age by choosing from the categories “20–24”, “25–29”, “30–34”, … “55–59” and “60+”.

We provide a pdf copy of the original online version of the full questionnaire which was developed for this study as Additional file 1.

### Optimizing the response rate

To optimize the response rate, the PI sent a briefing email to all members of the target list in advance of the official invitation to participate in the survey. This email briefed recipients on the purpose of the study and advised them that details of the funded project supporting this study would be made available by means of a customized survey link within the above invitation.

We kept the survey open over the period July 2013 to August 2014 and made provision for respondents to return to unanswered questions so as to allow for busy schedules and the need to verify information which was not immediately available. The PI sent regular reminders to non-participants and to those who had only partially completed the questionnaire. In each case, as with the initial invitation, potential respondents were reminded of the importance of their responses in contributing to an evidence-base for driving curriculum change irrespective of their own perceived level of expertise in statistics. This was combined with a clear emphasis concerning the availability of the option ‘don’t know’ for each listed statistical topic. These steps were taken to avoid any misconception among potential respondents of being unqualified to respond and any associated non-response bias.

### Data preparation

For the survey question pertaining to nature of employment, we merged response categories to form all possible combinations of choices made by respondents, including single categories and multiple categories. This resulted in the seven categories *Academic Research*; *Academic Teaching*; *Clinical Practice*; *Academic Teaching & Academic Research; Clinical Practice & Academic Research; Clinical Practice & Academic Teaching* and *Clinical Practice*, *Academic Teaching & Academic Research*. This was in order to more fully capture an individual’s employment status. For the purpose of summarizing our key findings and aligning these with our intended mixed model analyses, we also merged a) the option comprising *carry out the procedure or calculate the statistic(s) using appropriate data* as a sole competency with b) the option *both of the above*, comprising *both* the latter competency and *understand the theory*. We then assigned the abbreviated title *includes practice* to the resultant category. Also, we merged the remaining response categories to form the complementary category *does not include practice*. We in turn defined the response variable for our mixed model analyses as a binary variable with categories *includes practice* and *does not include practice*.

The classification of clinical specialisms provided by respondents (Q. 12) was informed both by a previous study involving medical graduates [11] and by a thematic approach whereby new specialisms (“the codes”) were derived retrospectively from responses (Additional file 2).

### Statistical analysis

We used IBM SPSS (v. 22) for graphical exploration of data, for generation of frequencies and percentages for inclusion in tables, and for graphical presentation of data. For mixed model analyses and corresponding assumptions testing and model comparisons we used the software R (v. 3.4.0, The R Foundation for Statistical Computing). Additionally, we used histograms and the Shapiro Wilks and Kolmogorov-Smirnov tests as tests of Normality to inform the appropriate choice of summary statistic for estimating length of time spent as an educator of medical undergraduates of the UoE (Q. 8).

To provide a first impression of the relative importance of topics, we ranked them in descending order of magnitude according to the percentage of participants for each topic who chose one of the two response options represented by our category *includes practice* as defined above (column 4 of Table 3). This was particularly important given that previous literature had dismissed or given little weight to the role of the medical graduate as a producer of statistics [12, 13].

We used a generalized linear mixed model (GLMM) principally to represent the role of *statistical topic* (*TOPIC*) as an explanatory variable for whether a response falls under the category *includes practice*. The dependent variable for this model was the binary variable with categories *includes practice* and *does not include practice*. We included fixed effects for *TOPIC* and nature of employment (*EMPLOYME*) and a random intercept for the respondent identifier, *RESPID* (which ranged over the survey respondents). The technical details of the model building process are provided in Additional file 2. For subsequent hypothesis testing, we assumed a *p*-value of less than 0.05 as an indicator of statistical significance. Our mixed model was also designed to take into consideration the hierarchical nature of the data structure, with individual responses (*includes practice* or *does not include practice*) at level one nested separately within each of *RESPID*, *TOPIC* and *EMPLOYME* at level two. Recognition of this structure was essential in avoiding overstatement of statistical significance [14].

For comparative purposes, the reference categories assumed for *TOPIC*, *EMPLOYME* and our above dependent variable were *Critical appraisal*, *Clinical practice* and *does not include practice*, respectively. Using these reference categories, we obtained odds ratios to represent the likelihood of a respondent having chosen a response option which included practice rather than one that did not include practice according to statistical topic and nature of employment.

We assessed the statistical significance of each odds ratio using a two-tailed t-test [15] and determined corresponding 95% CIs.

The associated null hypotheses were that the odds of selecting a response option of the type *includes practice* is identical for: a) the given statistical topic and *critical appraisal* and (separately) b) the given nature of employment category and *clinical practice*.

We intended the odds ratios involving topics to complement the rank ordering of topics and to provide an indication for different statistical topics of how important respondents considered the practice of that topic to be by comparison with critical appraisal. This was to allow a more balanced interpretation of the data than that forthcoming solely from the raw percentage data. We also used an omnibus test for the overall effect of each fixed effect (Additional file 2). For each of *TOPIC* and *EMPLOYME*, this involved testing the null hypothesis that the regression coefficients for the different categories of the independent variable were all equal to zero.

## Results

### Exclusions and determination of response rate

Three hundred and thirty-eight persons responded to the survey of whom five were excluded for the purpose of this study. Of these five, three had indicated (Q. 4) that they had never taught medical undergraduates of the UoE. In each case, we confirmed the accuracy of this response via the corresponding free text response to Q. 10 on engagement with students and involvement in their learning. Another respondent had specified their educational role as one not directly involving medical undergraduates and the remaining person had indicated that they were not a medical graduate. For the purpose of analysis, we retained a further respondent who had indicated that they had never taught medical undergraduates of the UoE as, based on their response to Q. 10, it was clear that they supervised medical undergraduates, and this type of interaction with students had been included in the definition of educator that we had offered previously. Of the remaining 333, we excluded a further 55 respondents since they had completed only the initial employment question (Q. 1) and had therefore made no contribution to questions on their experiences as educators to confirm their eligibility as respondents or to the key question (Q. 2) on statistical learning needs. Our results pertain to the remaining 278 respondents out of a target population of 462 respondents, corresponding to a response rate of 60.2%. These respondents represented over at least 77 unique clinical specialties. For completeness, we list the distribution of specialties represented by respondents in Table 1, where they are grouped under general headings purely for ease of reference.

**Table 1:** Existing clinical specialties for survey respondents

Specialty (frequency) ^a^	Total
*Allergy, Infectious diseases and virology*	9
Allergy (1), Infection control (1), Infections in haematology (1), Medical microbiology (4), Virology (2)	
*Critical and intensive care*	9
Critical care (7), Intensive care (2)	
*Diabetes and Endocrinology*	14
Diabetes (7), Endocrinology (7)	
*Gastroenterology, Hepatology and Nutrition*	9
Gastroenterology (6), Hepatology (2), Nutrition (1)	
*Obstetrics, gynaecology and neonatology*	27
Fetal medicine (1), Genitourinary medicine (1), Obstetrics and gynaecology (18), Neonatology (2), Reproductive Health (1), Reproductive medicine (2), Sexual health (2)	
*Oncology*	6
Gynaecological oncology (1), Oncology (4), Respiratory oncology (1)	
*Paediatrics and child health*	15
Community child health (1), Paediatric haematology (2), Paediatric neurology (2), Paediatric respiratory medicine (4), Paediatrics (6)	
*Pathology*	8
Clinical biochemistry (1), Pathology (7)	
*Psychiatry*	40
Psychiatry (13), Adult psychiatry (14), Child and adolescent psychiatry (5), Forensic psychiatry (1), Neuropsychiatry (1), Old age psychiatry (6))	
*Surgery*	42
Breast surgery (4), Cardiothoracic surgery (1), Colorectal surgery (4) General surgery (9), Head and neck surgery (1), Hepatobiliary surgery (3), Neurosurgery (1), Orthopaedic surgery (7), Plastic and reconstructive surgery (2), Spinal surgery (1), Transplant surgery (3), Urology (3), Vascular surgery (3)	
*Other*^b^	142
Acute medicine (7), Addiction medicine (3), Alcohol and substance misuse (2), Anaesthesiology (19), Cardiology (6), Clinical genetics (1), Clinical Pharmacology (2), Dermatology (3), ENT (2), General medicine (1), Geriatric medicine (8), Haematology (2), Neurology (5), Ophthalmology (2), Pain management (6), Palliative medicine (2), Patients with learning disabilities (1), Primary healthcare (14), Public Health (6), Clinical radiology (6), Rehabilitation (4), Renal medicine (4), Respiratory medicine (6), Rheumatology (3), Sleep medicine (1), Sport and exercise medicine (1), Stroke medicine (4), Toxicology (1), Transplant Medicine (1), None (19)	
Total	321

^a^Frequencies pertain to the number of occurrences of the corresponding specialty across free text responses to Q. 12 of the study questionnaire. As respondents were required to list all of their existing specialties, the total frequency exceeds the number of respondents

^b^The general category ‘*Other*’ is used to refer to those specialties which were identified as not falling under broader clinical categories during the classification of respondent data

### Demographics

**Fig. 1:**
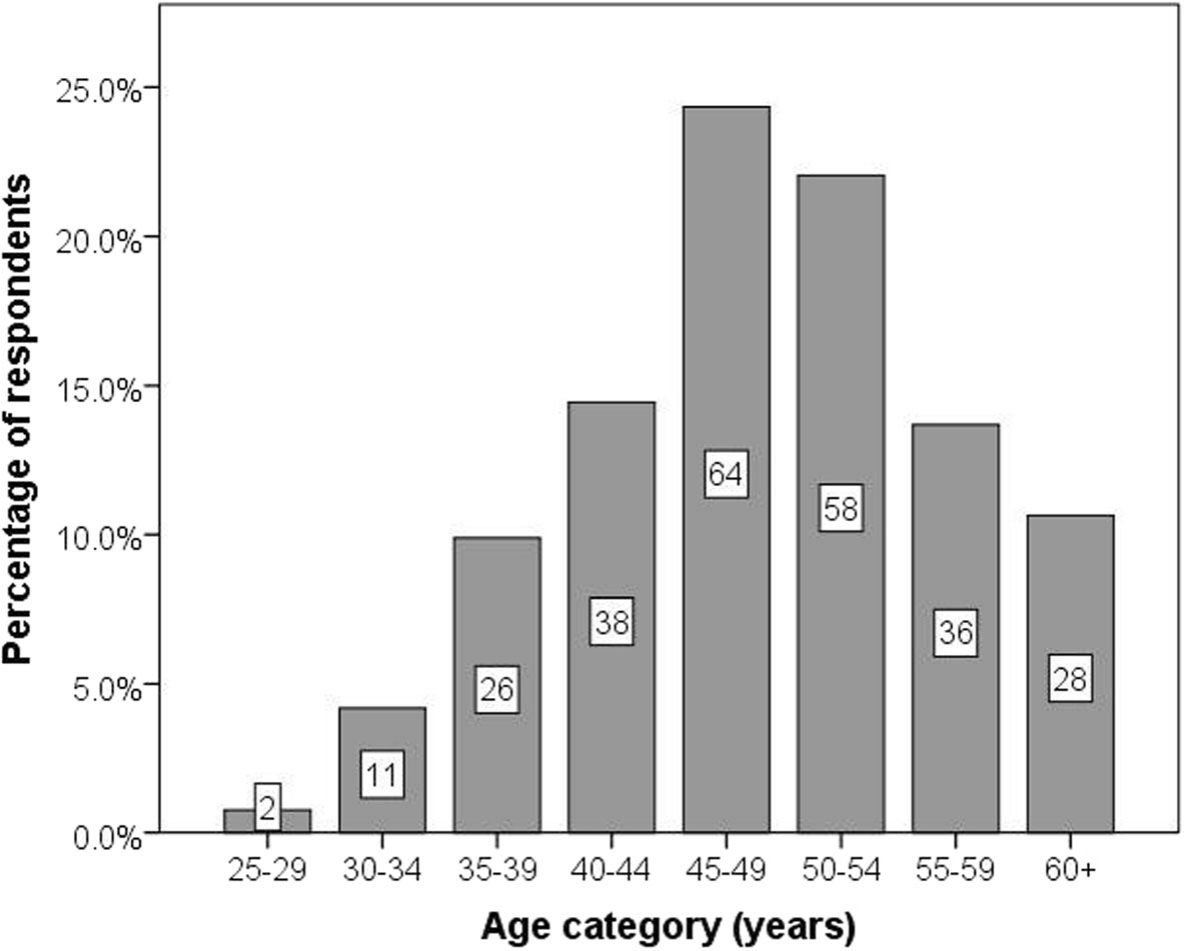
Age distribution of respondents

Of the 278 respondents included in the analyses, 263 (94.6%) provided data for age. The distribution of age categories is presented in Fig. 1.

For the 250 (89.9% of) respondents for whom adequate data were forthcoming on time spent as an educator of medical undergraduates of the UoE, the median time was 12.3 years, with a corresponding range of 0–49.9 years.

All 278 respondents provided data on nature of employment (Q. 1). In Table 2, we provide the distribution of respondent nature of employment.

**Table 2:** Frequency distribution for nature of employment of respondents

Employment Category	Frequency	Percentage
Academic Teaching	5	1.8
Academic Research	3	1.1
Clinical Practice & Academic Teaching	55	19.8
Clinical Practice & Academic Research	10	3.6
Academic Teaching & Academic Research	6	2.2
Clinical Practice, Academic Teaching & Academic Research	94	33.8
Clinical Practice	105	37.8
Total	278	100

In Table 3, we summarize the responses on competencies in statistics and probability that medical schools need to provide within the undergraduate medical curriculum to ensure thorough preparedness of new medical graduates for clinical practice. For ease of presentation, we have classified the statistical topics presented to respondents under general categories. These categories are non-unique and should not be interpreted as a basis for further analyses.

**Table 3:** a - e Relative frequency (as %) of medical graduate responses on competencies in statistics and probability that medical schools need to provide

Topic	n	rank	Required competency					
			Includes practice	Practice only	Theory and practice	Theory only	Neither	Don’t know
*a. Data visualization, Data preparation and manipulation* and *Software used for statistics*								
*Data visualization*								
*Graphical presentation of data:*	274	1	**84.3**	32.8	51.6	14.2	1.1	0.4
*Cross tabulating frequencies or percentages:*	273	23	**37.4**	15.0	22.3	28.9	16.1	17.6
*Survival analysis:*	277	39	**20.9**	5.4	15.5	65.0	7.2	6.9
*Forest plots:*	274	45	**15.0**	3.6	11.3	51.8	17.5	15.7
*Receiver operating characteristic (ROC) curves:*	275	46	**13.6**	2.6	11.0	45.4	18.3	22.7
*Cluster analysis:*	277	51	**6.1**	1.1	5.1	52.7	26.7	14.4
*Time series analysis:*	272	52	**5.9**	2.2	3.7	36.4	27.6	30.1
*Data preparation and manipulation*								
*Arranging data in spreadsheets for statistical analysis:*	278	2	**81.7**	25.5	56.1	14.4	2.2	1.8
*Working with subsets of the original data - filtering data:*	274	27	**33.2**	13.9	19.3	38.0	15.3	13.5
*Merging similar datasets:*	273	42	**17.9**	6.2	11.7	38.8	27.5	15.8
*Types of response data:*	272	49	**9.6**	2.9	6.6	41.5	17.3	31.6
*Software used for statistics*								
*Using Excel for statistics-tips and warnings*	270	7	**64.1**	26.3	37.8	23.0	9.3	3.7
*Getting to know the fundamentals of a statistical package such as SPSS:*	274	11	**57.3**	27.0	30.4	22.6	14.6	5.5
*b. Ratios, rates and proportions*, *Summarising data* and *Probability theory*								
*Ratios, rates and proportions*								
*Sensitivity, specificity and positive and negative predictive values:*	272	4	**75.0**	26.1	48.9	24.3	0	0.7
*Comparing a study cohort with a general population:*	274	22	**38.3**	13.5	24.8	56.6	4.4	0.7
*Statistical risk estimates:*	272	38	**23.0**	5.2	17.8	62.6	6.3	8.1
*Summarising data*								
*Simple descriptive (or summary) statistics:*	272	5	**69.9**	24.6	45.2	21.7	5.5	2.9
*Confidence intervals:*	278	6	**65.1**	20.9	44.2	34.5	0.4	0.0
*Summarising and analysing missing data:*	267	43	**17.6**	4.9	12.7	50.2	20.6	11.6
*Probability theory*								
*Laws of probability:*	278	12	**48.9**	12.2	36.7	46.4	3.2	1.4
*Concepts and rules of probability:*	276	25	**34.5**	10.2	24.4	61.1	2.9	1.5
*c. Reporting statistics* and *Avoiding bad practice in statistics and exploring study design*								
*Reporting statistics*								
*Presenting the findings and conclusions of statistical hypothesis tests:*	276	8	**63.4**	20.7	42.0	28.6	5.4	3.3
*Statistical significance, statistical power and some facts about p-values:*	275	10	**58.5**	18.9	39.6	39.6	0.7	1.1
*Valid reporting and interpretation of statistical findings:*	274	14	**46.0**	12.4	33.5	48.4	3.6	2.2
*Statistical effect sizes:*	274	15	**44.9**	11.3	33.6	51.5	2.2	1.5
*Avoiding bad practice in statistics and exploring study design*								
*Principles of good study design:*	275	16	**42.6**	14.2	28.4	5.3	1.8	0.4
*Sample size calculations:*	272	18	**41.2**	14.0	27.2	47.1	8.5	3.3
*Randomization:*	275	20	**39.6**	13.1	26.5	59.3	0.7	0.4
*Misuse of statistics: some statistical blunders and phenomena to look out for in published literature:*	274	21	**39.4**	10.6	28.8	52.9	5.1	2.6
*Different types of study design:*	275	26	**34.2**	9.1	25.1	64.7	0.4	0.7
*Statistical aspects of clinical trials*:	272	32	**29.4**	6.3	23.2	65.8	2.9	1.8
*Retrospective power calculations* versus *examination of confidence intervals:*	275	47	**12.7**	2.5	10.2	48.7	21.5	17.1
*Cross-over trials:*	271	48	**11.8**	2.6	9.2	70.5	10.3	7.4
*d. Procedures explicitly requiring statistical hypothesis testing* and *Assessing agreement, consistency and correlation*								
*Procedures explicitly requiring statistical hypothesis testing*								
*Tests of normality:*	274	17	**41.7**	11.3	29.9	43.8	8.0	6.9
*Simple linear regression analysis:*	278	19	**39.6**	14.4	25.2	43.9	11.2	5.4
*Hypothesis tests for a single group of continuous data:*	269	24	**35.3**	10.4	24.9	35.4	13.0	16.4
*One-tailed* versus *two-tailed hypotheses tests:*	271	28	**32.8**	9.6	23.2	48.7	9.2	9.2
*Hypothesis tests for categorical data:*	271	30	**32.1**	8.5	23.6	35.8	14.0	18.1
*Hypothesis tests for comparing two groups of measurement or ordinal data:*	270	33	**29.3**	5.9	23.0	31.5	17.4	21.9
*Analysis of variance (ANOVA)*:	270	36	**27.0**	8.5	18.5	45.6	15.2	12.2
*Multiple linear regression analysis:*	278	41	**18.3**	6.8	11.5	58.3	16.2	7.2
*Analysis of covariance (ANCOVA)*:	269	44	**16.7**	5.9	10.8	42.4	23.0	17.8
*Tests of homoscedasticity (or, ‘equality’ of variance):*	269	50	**6.7**	1.5	5.2	30.5	30.1	32.7
*Assessing agreement, consistency and correlation*								
*Correlation coefficients –linear and non-linear:*	271	34	**29.2**	8.1	21.0	53.1	10.0	7.7
*Statistical indices for measuring levels of agreement and consistency:*	274	37	**27.0**	7.3	19.7	55.1	9.9	8.0
*Assessing agreement between two methods of measurement:*	268	40	**18.7**	5.6	13.1	48.9	16.4	16.0
*e. Allied topics* and *Critical appraisals and systematic reviews*								
*Allied topics*								
*Understanding the proper meaning of an audit:*	275	3	**81.5**	29.1	52.4	16.4	1.1	1.1
*Designing survey questions to support valid statistical analyses:*	271	13	**48.0**	15.9	32.1	36.2	10.0	5.9
*Health-related data sources:*	272	29	**32.7**	9.9	22.8	45.2	7.7	14.3
*Representing socioeconomic status:*	278	35	**29.1**	6.5	22.7	58.6	6.1	6.1
*Critical appraisals and systematic reviews*								
*Conducting critical appraisals:*	271	9	**62.0**	21.0	40.2	30.3	4.1	4.4
*Systematic reviews:*	267	31	**31.5**	8.2	23.2	65.5	2.2	0.7

Note. The column header ‘n’ denotes the number of responses for the given topic, while ‘Practice only’, ‘Theory and practice’ and ‘Theory only’ are abbreviations used for the listed response options ‘carry out the procedure or calculate the statistic(s) using appropriate data’, ‘both of the above’ and ‘understand the theory only’, respectively. Correspondingly, column 4 is formed by combining columns 5 and 6. The entries in this column are included in bold for ease of reference when identifying how the corresponding ranks were obtained in column 3. Percentages are row percentages with the denominator in the calculation pertaining to the number of persons who responded for the listed topic, inclusive of those who responded, ‘Don’t know’. Percentages in the main text of this paper are obtained from combining original frequencies for two response options (e.g. those pertaining to columns 6 and 7). They may therefore differ slightly from those obtained by simply adding the corresponding percentages in the table. This is to avoid rounding errors

In Additional file 3: Table S1, we also include the ranks, frequencies and percentages presented in Table 3a - e without sub-division of content by general topic. This is with the understanding that a single table of statistical topics, ordered according to ranks, could be particularly valuable in allowing flexible use of our study findings for course design.

Comparison of the 105 (37.8% of) respondents who chose exclusively clinical practice as the nature of their employment with the remaining 173 (62.2% of) respondents (Table 2), whom we combined under the category *Other*, revealed a tendency for respondents from the former group to be less likely to select a competency involving the practice of statistics for a given statistical topic. This was particularly evident from the relative percentages of respondents selecting this type of competency for the topics *Presenting the findings and conclusions of statistical hypothesis tests* (Clinical practice: 48.6%, Other: 70.5%), *Simple descriptive (or summary) statistics* (Clinical practice: 53.3%, Other: 77.5%), *Graphical presentation of data* (Clinical practice: 72.4%, Other: 89.6%), *Hypothesis tests for a single group of continuous data* (Clinical practice: 19.0%, Other: 43.4%), *Hypothesis tests for comparing two groups of measurement or ordinal data* (Clinical practice: 15.2%, Other: 36.4%) and *Analysis of variance (ANOVA)* (Clinical practice: 14.3%, Other: 33.5%).

### Mixed model analyses

Using the two-level mixed effects model described earlier, we found that nature of employment and statistical topic were highly significant predictors of choice of the practice of statistics as a required competency ((F = 3.777, p < 0.0005) and (F = 45.834, p < 0.0005), respectively). Assuming *Clinical Practice* as the reference category, the odds ratios and corresponding confidence intervals for nature of employment as a predictor of the response category *includes practice* are provided in Table 4.

**Table 4:** Odds ratios for selection of statistical learning needs response option which includes practice according to employment category

Employment Category	Odds ratio	95% CI	*p*-value
Academic Teaching	0.79	(0.15, 4.13)	0.777
Academic Research	4.01	(0.49, 32.64)	0.194
Clinical Practice & Academic Teaching*	1.91	(1.04, 3.49)	0.036*
Clinical Practice & Academic Research*	3.85	(1.77, 12.62)	0.026*
Academic Teaching & Academic Research*	6.64	(1.46, 30.13)	0.014*
Clinical Practice, Academic Teaching & Academic Research**	2.84	(1.70, 4.74)	0.000**
Clinical Practice *(reference category)*	–	–	–

Note. *p*-values are rounded to 3 decimal places. ‘*’ denotes ‘*p* < 0.05’ and ‘**’ denotes ‘*p* < 0.0005’

**Fig. 2:**
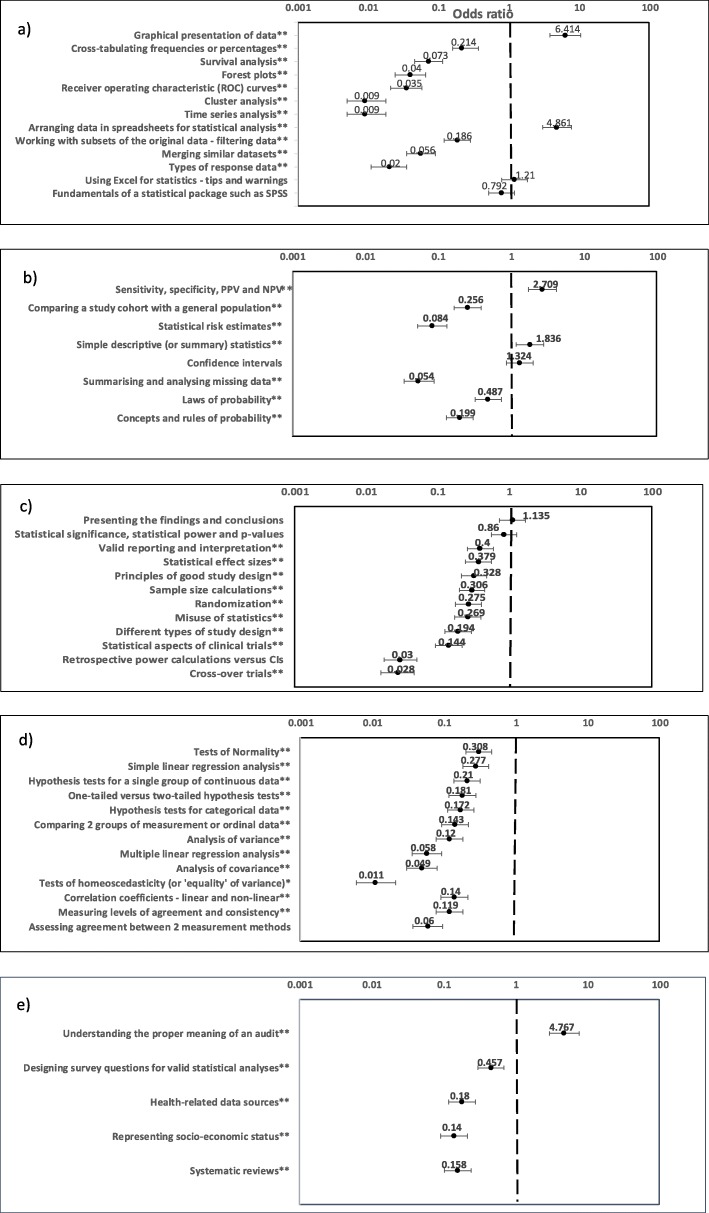
Odds ratio and corresponding 95% CI for the binary dependent variable with categories *includes practice* and *does not include practice* according to topic in statistics and probability. ‘*’ denotes ‘*p* < 0.05’ and ‘**’ denotes ‘*p* < 0.0005’. The corresponding variable reference categories are *does not include pratice* and *critical appraisal*, respectively. The abbreviated topic names listed in parts **a**-**e** of this figure correspond to those listed in parts **a**-**e**, respectively, of Table 3

In Fig. 2, we report the odds ratios for the binary dependent variable for our mixed model according to statistical topic.

### Further statistical topics

The response data for the query (part 2 of Q. 2) on what was missing from the available list of statistical topics were not sufficiently comprehensive to merit a grounded theory approach to content analysis. However, they yielded some valuable pedagogical ideas for delivery of statistical learning. For these reasons, they are presented in Additional file 4.

## Discussion

### Integration of clinical and statistical learning

The clear link to clinical practice in the stem of Q. 2 reflects our tacit assumption that both theory and practice in statistics should be delivered through clinically contextualized examples and that this should extend to the choice of datasets for analysis of data. This is in keeping with the perspective of Mustafa [16], who views “the ability to link statistics and real-world situations” as a competency which ought to be developed generally in the teaching of statistics to non-specialists, of Singer and Willet [17], who favour the choice of real-life over artificial datasets, of Sahai and Ojeda, who recommend that the account of data collection “should flow from the medical question” [18] and the recommendation in the current GAISE guidelines to “Ground activities in the context of real data with a motivating question” [19]. However, noting the extra demand on resources for developing the competency *carry out statistical procedures and calculations using appropriate data*, we have also sought to gather *topic-specific* information on the need for this competency (in addition to or as an alternative to that of understanding the theory) in preparation for clinical practice.

### Demographics

The age distribution of respondents is slightly skewed to the left (Fig. 1). This is unexceptional, given the likely requirement of more junior doctors to be enrolled in advanced training, such as specialist registrar programmes, in order to gain entry to their chosen speciality.

The findings from this study are supported by the target group representing a wide range of clinical specialties (Table 1), although it is of interest to note (Table 2) that of the 278 eligible respondents, all of whom had confirmed or provided evidence of their status as educators (Q.’s 4 and 10), 118 (42.4%) did not perceive academic teaching as an eligible choice of descriptor for the nature of their employment. This may reflect perceptions among medical graduates of the status of teaching within their profession.

### Relative popularity of statistical topics

Table 3 illustrates that respondent views concerning the roles of consumer and producer of statistics vary according to choice of statistical topic.

As one might expect from the nature of the topic, the response data for *conducting critical appraisals* is strongly weighted *against* understanding the theory only in favour of including practice (62.0% of respondents). (column 4, Table 3e) As Table 3 reveals more generally, however, this is not to the exclusion of statistical topics that are meaningful in their own right.

The topic *Graphical Presentation of Data* (Table 3a) was the most popular in terms of responses of the type *includes practice* (84.3% of respondents). Through focusing on the need for physicians to correctly interpret medical literature to keep abreast with the developments in their field [18], respondents might have considered the need to competently interpret graphs in clinical papers as adequate, leading them to opt for *understand the theory only*. Thus, the above finding is instructive in determining future learning needs.

The popularity (rank = 2, 81.7% of respondents) of the topic *arranging data in spreadsheets for statistical analysis* (Table 3a) resonates with previous work, where provision of a comprehensive data preparation tutorial is recommended as an exemplar for counteracting psychological barriers to learning in statistics [10]. The topic *Understanding the proper meaning of an audit* (Table 3e) was almost equally popular (rank = 3, 81.5% of respondents). This suggests that the practice of carrying out an audit is recognized, at least by the respondents for this study, as a routine quality assurance activity that is integral to clinical practice.

We found the topic *confidence intervals* (Table 3b) to be more popular (rank = 6, 65.1% of respondents) than any of the topics listed under ‘Procedures explicitly requiring hypothesis testing’ (Table 3d). This is consistent with recommendations in the literature. Over three decades ago, Gardner and Altman [20] were instrumental in defending the greater usefulness of confidence intervals by comparison with findings from hypothesis tests. Efforts have continued thereafter to keep this perspective in view, even to this present day as debates over the future of *p*-values continue [21].

The popularity of understanding the theory for the topic Misuse *of statistics: some statistical blunders and phenomena to look out for in published literature* (81.8% of respondents, Table 3c) may be best carried forward into teaching practice through development of this topic as a common theme for *all* statistical learning opportunities. Such teaching could be enhanced both by topical examples from the media, as suggested in our respondent free text data (Additional file 4) and in the educational literature [18], and by recognition of the critical place of Bayes’ Theorem in understanding diagnostic statistics [7, 18, 22]. This includes in supporting critical appraisal, noting Simpson’s personal viewpoint that, “Without an appreciation of the ways in which statistics can be used and abused, students will find it difficult to understand and critically appraise the literature in their subject” [22].

In preparation for clinical practice, this extends to recognition of lack of statistical transparency in leaflets which physicians receive directly from the pharmaceutical industry. Busy clinicans with inadquate statistical training are likely to be poorly equipped to recognize statistical clues that the results have been “systematically distorted or important details omitted;” [7] rather, the leaflet may find its way into the patient-doctor consultation based on aesthetic appeal and the persuasive nature of its content.

Furthermore, the need expressed elsewhere for cultivating awareness of confounding and multivariable relationships in statistics education [19] could be conveniently met through the above theme, including through inclusion of stratification and Simpson’s paradox [19]. Clearly, a sound conceptual understanding of *cross-over trials* (Table 3c), was recognized as particularly relevant to clinical practice, noting that the above topic proved to be the most popular for the competency *understanding the theory only* (70.5% of respondents).

Concerns about errors in calculations and personal accountability when communicating risk to patients may partly explain why a strong majority (approximately 63%) of respondents indicated a preference for understanding the theory only in relation to statistical risk estimates. (Table 3b).

The topics *ANOVA* and *Statistical indices for measuring levels of agreement and consistency* (Table 3d) attained the relatively low ranks of 36 and 37, respectively under competencies of the type *includes practice*. These topics encompass a wide range of designs, including repeated measures designs, and estimation of agreement and correlation according to these designs. This may not have been apparent to all respondents and including *repeated measures designs* in our list of statistical topics may therefore have proved helpful. This type of terminology might have resonated well with experiences of clinical practice, noting that clinicians frequently take multiple readings over time to monitor effectiveness of treatments without necessarily engaging in clinical trials research.

### Observations from mixed model analysis

The statistically significant odds ratios, ranging from 1.91 to 6.64 in Table 4, are supportive of a strong employment effect. It is particularly noteworthy that, by comparison with indiviuals who opted for *Clinical Practice* only, respondents from other employment categories tended to be more likely to choose a response option which included pratice. This suggests that aspects of a respondent’s employment other than their own clinical practice – namely, academic teaching and academic research – influenced them to favour practical training in statistics or probability as an aspect of the learning needs of medical students in preparation for clinical practice. This is consistent with the preliminary findings prior to mixed model analysis on comparing choices of competencies by respondents from clinical practice only with those of all other respondents.

Table 4 also reveals more specifically that the above relationship was most prominent for respondents who identified academic research as at least a component of the nature of their employment. Candidates falling under the employment category *Academic Teaching & Academic Research*, with the highest odds ratio, may have used their own research in their teaching to prepare medical undergraduates for clinical practice. This is particularly plausible, given the increased popularity of research-informed teaching within higher education over recent years.

However, it is important to acknowledge the small group sizes (Table 2) and correspondingly wide CIs (Table 4). These reflect low accuracy in the estimation of the true odds ratio and are unsupportive of sub-group analyses. By contrast, *Clinical Practice, Academic Teaching & Academic Research* is a dominant category (38.1% of respondents) relative to all the other *nature of employment* categories, which may explain the elevated level of statistical significance relative to the other employment categories for which odds ratios are listed. Nevertheless, from hypothesis testing, it is also clear that *overall*, nature of employment (as defined by the response categories in Table 2) is a highly significant predictor of choice of the binary response category *includes practice.*

For those topics which are close to *Critical appraisal* in rank (Table 3), there is a lack of evidence using mixed model analysis that they are significantly less or more important than critical appraisal as candidate topics for the development of student competency in the practice of statistics and probability (Fig. 2). Such topics include those falling under *Software used for statistics* (last two categories in Fig. 2a) and *Presenting the findings and conclusions of statistical hypothesis tests* and *Statistical significance, statistical power and some facts about p-values* (first two categories in Fig. 2c), with the corresponding odds ratio being close to 1 in each case. By contrast, the two most highly ranking topics in Table 3, *Graphical presentation of data* and *Arranging data in spreadsheets for statistical analysis*, are estimated, respectively, to be over six times and almost five times more important than *Critical appraisal*, with a high level of statistical significance (Fig. 2a). The remaining topics which are found to be significantly more important than *critical appraisal* are *Sensitivity, specificity and positive and negative predictive values* (diagnostic statistics), *Simple descriptive (or summary) statistics* (Fig. 2b) and *Understanding the proper meaning of an audit* (Fig. 2e). The importance of descriptive and diagnostic statistics from the perspective of medical graduates is consistent with Simpson’s viewpoint, who in addition to placing a strong emphasis on diagnostic statistics in her own teaching of medical undergraduates to reflect the needs of clinical practice, recommends that, “Any introductory course should start with descriptive statistics… Without an understanding of variability, the rest of the course will be meaningless” [22]. Additionally, in considering clinically relevant content to include within the scope of descriptive statistics, it is helpful to note Sahai and Ojeda’s reference to the practical importance of percentiles for establishing cut-offs for defining normal ranges for biochemical and physiological measurements in patient diagnosis [18]. The latter example is a welcome reminder of the principle held more generally by teachers in service courses, that students must see the relevance of statistics to their chosen discipline [23] and, we would add, to their chosen profession.

More generally, our findings suggest that, while critical appraisal ought to have a prominent place within the undergraduate medical curriculum in teaching the practice of statistics, there are statistical topics which may need to take greater priority or be afforded equal priority in order to meet recognized needs for clinical practice.

### Strengths and limitations

While having a well-defined target group was critical in obtaining an accurate estimate of the response rate, the generalizability of our findings is likely to be limited by the restriction that respondents required to have had prior or current experience as educators of UoE medical undergraduates. Also, there were many potential impediments to completion of the study questionnaire by the target group. These included the tendency for statistics to be unpopular among non-specialists, the competing demands on time of potential respondents in relation to their teaching, research and clinical commitments, and the comprehensive nature of the questionnaire. However, more recent interest in the analysis of big data within Medicine since the timeframe for the survey may have led to changed views among physicians concerning student learning needs in statistics, including an upward trend in the proportion who support training in the analysis of data.

Further, in presenting statistical topics to respondents, richer findings might have been obtained by classifying many of the topics listed under *Avoiding bad practice in statistics and exploring study design* in Table 3c) according to different types of study design, including cluster-randomized trials and pragmatic randomized controlled trials rather than singling out cross-over trials as a specific type of trial design. Additionally, had this been a multi-institutional study, the list of statistical topics in the questionnaire might have differed dependent on experiences of contributors as statistical educators and the response data might have been influenced by inter-institutional variation in clinical specialties for respondents.

We cannot guarantee the absence of non-response bias, although some evidence concerning this type of bias would have been forthcoming had we asked potential respondents about their statistical background. Potential respondents may not have read the relevant content in briefing emails and the text adjoined to the stem of Q. 2 regarding both the inclusive nature of the study and the availability of the response option ‘don’t know’. Also, it is possible that choice of the latter option was based in some cases, on a lack of understanding of what the listed statistical topic encompassed. For example, the surprisingly high percentage (32%) of respondents who chose this option for *types of response data* may have included a considerable number of individuals who would have found the wording *data type of variables* for this topic less ambiguous.

Nevertheless, the high level of granularity in available statistical topics for respondents, reliance on medical graduate *experiences* of clinical practice and the favourable response rate, for our survey, ought to strengthen the current evidence base for choice of statistical content in designing undergraduate medical curricula. This is particularly evident given the preponderance in the educational literature of opinion pieces arising from statistical educators concerning the statistical learning needs of medical undergraduates [13, 18, 22, 24–26], with other work specifically focused on “the importance of statistical competencies for medical research learners” [27].

It is important to appreciate that this is the first high-resolution study examining the statisical learning needs of undergraduate medical students specifically in preparation for clincial practice and in turn, defending the role of medical graduates as producers of statistics. In designing the survey, care was taken to ensure that responses on choice of statistical competencies according to topic were not arbitary, with clinicans being asked to “use [their] own experience as a medical graduate”. However, we also emphasize that we were unable to provide anecdotal and specialty-focused evidence from our study regarding how statistical learning enhances clinical practice. Noting that there is a corresponding gap in the current medical educational literature, this would be a highly valuable area to explore, ideally through qualitative research approaches, including focus groups and semi-structured interviews, with snowball sampling of participants [28]. Such research could generate case studies for clinical practice where medical graduates use statistics within their own specialties or recognize the need for better statistical training at the undergraduate level to prepare them to carry out their clinical decisions more competently. This would help in strengthening the evidence base from the current study. Ideally, such case studies could in turn be carried forward into undergraduate teaching, thus enhancing the appreciation of the relevance of statistics among medical students and educators, alike.

### Considerations for future course design

In designing statistical learning opportunities for medical undergraduates which reflect the needs of clinical practice, negotiating *adequate* space within existing clinical modules in a crowded curriculum may prove impractical. Designing standalone statistical modules involving clincally contextualized case studies is an additional route to follow. However, this approach presents challenges which invite strategic planning.

The development of modules is labour intensive. If such modules are made available during term time, uptake and engagement may be limited by the pressures of curricular deadlines and assessment. It may therefore be a viable option to deliver Massive Open Online Courses (MOOCs) throughout the year, but with the recommendation that students consider completing the MOOCs over the summer vacation. The latter recommendation is consistent with exisiting practices across different universities on an international scale, where students have the opportunity to participate in university-led internships, research projects, summer schools, and university award schemes grounded on the development of transferable skills. The above option could support distance learning by provision of downloadable institutionally licensed statistical software. To identify authentic data-sets for teaching and assessment purposes, instructors may benefit from reaching out to colleagues and from exploration of institutional data repositories and open data journals. Collaborative learning among students may be employed as a useful means of addressing the challenges of providing unique datasets and timely comprehensive feedback to a large cohort of students. As noted in the current GAISE guidelines [19, 29], collaborative learning can enhance student skills in communicating statistics, which is also of relevance to clinical practice (Additional file 4).

## Conclusions

The research-informed findings from this study provide a good basis for improving learning in statistics and probability in the undergraduate medical curriculum at the UoE. The rich survey response data indicate that the need in clinical practice to carry out the procedure or calculate the statistic(s) using appropriate data is well recognized. Furthermore, these data suggest that competencies in statistics and probability which medical graduates require for their own clinical practice span a wide range of statistical topics and are not restricted to understanding the theory. Such findings contrast with the viewpoint expressed elsewhere that, “medical statistics courses should focus on critical appraisal skills rather than on the ability to analyse data” [12]. They also at least outdate those of Marks, who in contrasting clinicians with researchers, states that the former “have no need for analytical abilities, either by computer or manually” [13].

Further, we have identified ideas both from the broader literature on statistical education and from current educational practices to enrich the choice of subject content and style of delivery on carrying forward the findings of our study. This has included students completing modules during their summer vacation through collaborative learning as an approach to circumventing resource and timetabling issues experienced within undergraduate medicine on an international scale.

## Supplementary information

**Additional file 1.** Survey for Medical Graduates on Statistical Learning Needs (pdf copy). This is a pdf copy of the original online version of the study questionnaire.

**Additional file 2.** Further methodological details. Additional file 2 provides further methodological details concerning the procedures for data preparation, model building and model selection used for presentation and statistical analysis of the response data for this study.

**Additional file 3: Table S1.** Table S1 is a single table including the ranks, frequencies and percentages presented in Table 3 without sub-division of content by general topic.

**Additional file 4.** Further topics in statistics and probability emergent from free text response data and the need for boundaries. Additional file 4 provides: a) a brief narrative synthesis of the free text comments arising from the second part of Q. 2 of the study questionnaire, pertaining to what was missing from the available list of topics and b) recommendations for approaches to teaching statistics suggested by the emergent themes associated with these free text responses.

## Data Availability

The anonymized datasets generated or analysed during this study are available from the corresponding author on reasonable request. They are not publicly available, as permission was not explicitly sought from survey respondents for use of their data in this way.

## Abbreviations

ANOVA: Analysis of variance
GLMM: Generalized linear mixed model
NHS: National Health Service
PI: Principal Investigator
