# Supplementary material for: Medical graduate views on statistical learning needs for clinical practice: a comprehensive survey
Source: BMC Med Educ. 2019 Dec 31;20:1. doi: 10.1186/s12909-019-1842-1 (PMC6937818; doi:10.1186/s12909-019-1842-1)
Supplement: Supplementary file 1 — Additional file 1. Survey for Medical Graduates on Statistical Learning Needs (pdf copy). This is a pdf copy of the original online version of the study questionnaire. [file 12909_2019_1842_MOESM1_ESM.pdf]

Appendix 1

Survey for Medical Graduates on Statistical Learning Needs (pdf copy of original online version)

1. Main purposes of survey

Dear Colleague

Thank you for your interest in completing this survey. The information you provide will prove extremely informative in defining a statistics curriculum for undergraduate medical students which is informed by the experiences of medical graduates. You will also be helping to address the aims of the project
Preparing medical students for self-directed learning in statistics: What should we expect of tomorrow's doctors?
which is currently funded under the University of Edinburgh Principal's Teaching Award Scheme. Please click on the project title above to access the project abstract.

The data collected from this study should also prove most useful in addressing future questions in educational research.

NB. If you are unable to access the project abstract via the linked title, above, please copy the following URL into your browser space:

http://www.cphs.mvm.ed.ac.uk/docs/PTAS_abstract.pdf .

Survey for Medical Graduates on Statistical Learning Needs (pdf copy of original online version)

2. Confidentiality

The data in its raw form will be kept exclusively by the University of Edinburgh. For the purpose of dissemination (including publication) of the research findings for the benefit of non-members of the project team your responses will be completely anonymized. However, you should feel most welcome to contact the Principal Investigator at any stage if you have any queries or concerns.

As a questionnaire respondent, you will be kept up-to-date with any publications which are forthcoming from this research.

Survey for Medical Graduates on Statistical Learning Needs (pdf copy of original online version)

3. Note of appreciation and contact details

In completing this survey, you are consenting to your data being used for the above purposes.

Thank you in advance for your kind co-operation.

Margaret MacDougall, Principal Investigator

Contact details for further information:
Dr Margaret MacDougall, Medical Statistician and Researcher in Education
Centre for Population Health Sciences, College of Medicine and Veterinary Medicine, University of Edinburgh Medical School, Teviot Place, Edinburgh, EH8 9AG

Tel: +44 (0)131 650 3211
E-mail: Margaret.MacDougall@ed.ac.uk

Survey for Medical Graduates on Statistical Learning Needs (pdf copy of original online version)

4. Using the survey

The survey questions commence on the next page. To assist you in completing the survey, some basic instructions are provided below.

You can progress to the next question by scrolling down if necessary and clicking the button 'Next' **at the bottom of the page**.

You can edit your responses to previous questions by clicking the button 'Previous' **at the bottom of the page**.

Please do not use your browser arrows to attempt to navigate between survey pages.

A progress bar is provided at the top of individual pages to indicate how far you have progressed through the survey.

To submit your responses, click 'Done' at the end of the survey **unless you wish to return to the survey later via the link provided in the invitation email**.

Survey for Medical Graduates on Statistical Learning Needs (pdf copy of original online version)

5. Nature of your employment

*** 1. Please select ALL options which describe the nature of your employment.**

- [ ] Clinical practice
- [ ] Academic research
- [ ] Academic teaching
- [ ] Other (please specify)

Survey for Medical Graduates on Statistical Learning Needs (pdf copy of original online version)

6. Statistical competences needed by medical graduates for clinical practice

*** 2. Please use your own experience as a medical graduate to identify those competencies in statistics and probability that medical schools need to provide within the undergraduate medical curriculum to ensure thorough preparedness of their new medical graduates for clinical practice.**

For each of the topics listed below, please select the most appropriate response.

The option 'don't know' is available for your use wherever appropriate.

Please also use the "What's missing?" box if you feel the list is incomplete, while specifying the corresponding drop-down menu option you would have chosen if the item had been listed. <u>The accuracy of your responses to this question is critical so please proof-check your responses to check you haven't skipped any topics. Thank you for your patience.</u>

Arranging data in spreadsheets for statistical analysis	
Representing socioeconomic status	
Laws of probability	
Getting to know the fundamentals of a statistical package such as SPSS	

Appendix 1

Simple linear regression analysis	
Multiple linear regression analysis	
Confidence intervals	
Statistical effect sizes	
Statistical indices for measuring levels of agreement and consistency	
Cluster analysis	
Systematic reviews	
Presenting the findings and conclusions of statistical hypothesis tests	
Survival analysis	
Comparing a study cohort with a general population	
Understanding the proper meaning of an audit	
Concepts and rules of probability	
Forest plots	
Conducting critical appraisals	

Appendix 1

Misuse of statistics: some statistical blunders and phenomena to look out for in published literature	
Principles of good study design	
Different types of study design	
Randomization	
Statistical risk estimates	
Valid reporting and interpretation of statistical findings	
Retrospective power calculations versus examination of confidence intervals	
Statistical significance, statistical power and some facts about p-values	
Working with subsets of the original dataset - filtering data	
Merging similar datasets	

Appendix 1

Summarizing and analyzing missing data	
Simple descriptive (or, summary) statistics	
Cross-tabulating frequencies or percentages	
Graphical presentation of data	
Tests of Normality	
Sensitivity, specificity and positive and negative predictive values	
Receiver Operating Characteristic (ROC) Curves	
Confidence intervals	
One-tailed versus two-tailed hypotheses tests	
Hypothesis tests for a single group of continuous data	
Hypothesis tests for categorical data	

Tests of homoscedasticity (or, 'equality' of variance)	
Hypothesis tests for comparing two groups of measurement or ordinal data	
Analysis of variance (ANOVA)	
Analysis of covariance (ANCOVA)	
Statistical aspects of clinical trials	
Correlation coefficients - linear and non-linear	
Cross-over trials	
Assessing agreement between two methods of measurement (continuous data)	
Time series analysis	
Using Excel for statistics: tips and warnings	
Types of response Data	
Sample size calculations	

Designing survey questions to support valid statistical analyses

Health-related data sources

What's missing?

3. Based on your response to the previous question, please provide concrete examples of your past or present use of statistics or probability in clinical practice. If there are instances where you did not have the pre-requisite skills in statistics or probability but recognized the need for these skills, please explain. There is not a requirement for you to get the statistical terminology exactly right if you feel unable to do so. However, your response to this question may drive curriculum change so please be as clear and comprehensive as possible.

Survey for Medical Graduates on Statistical Learning Needs (pdf copy of original online version)

7. Teaching of University of Edinburgh undergraduate medical students

N.B. The questions in this section specifically refer to your experience of teaching undergraduate medical students from the University of Edinburgh, not from any other university.

Please note that the term 'educator' can be interpreted broadly as covering a wide range of pedagogical roles, including 'learning facilitator' and 'research mentor'. You may also be involved in supporting student placement activities without being an employee of the University of Edinburgh. If you feel that your knowledge of the 'Edinburgh' undergraduate medical curriculum is limited, please still take time to respond. A 'don't know' option is provided for your use in all appropriate questions and this type of response matters too.

*** 4. Please choose the category which best describes your role as an educator of undergraduate medical students from the University of Edinburgh.**

- ○ Current
- ○ Previous
- ○ Never

Survey for Medical Graduates on Statistical Learning Needs (pdf copy of original online version)

8. Teaching of University of Edinburgh undergraduate medical students

*** 5. Can you think of any instances in your own teaching of undergraduate medical students of the University of Edinburgh where the integration of learning in statistics or probability with clinical learning would enhance the quality of the student learning experience?**

- ○ No
- ○ Don't know
- ○ Yes - where possible, please specify:

a) the Year(s) of study,

b) the particular clinical topic, corresponding contact person(s) and teaching or learning style(s) you recommend

and

c) in as much detail as possible in your own words, what areas in statistics or probability need to be included.

Survey for Medical Graduates on Statistical Learning Needs (pdf copy of original online version)

9. Teaching of University of Edinburgh undergraduate medical students

*** 6. Are there any specific learning contexts within the Edinburgh MBChB curriculum where it would be valuable to integrate online discussion forums with eLearning activities in statistics or probability during clinical problem solving or decision making?**

- No
- Don't know
- Yes - please specify in as much detail as possible

Survey for Medical Graduates on Statistical Learning Needs (pdf copy of original online version)

10. Teaching of University of Edinburgh undergraduate medical students

* 7. For the contexts which you have listed, would you be willing to share my role as tutor in:

	yes	no	maybe
setting problem-solving tasks for undergraduate medical students to address in strictly scheduled online discussions	◯	◯	◯
marking performance on these tasks	◯	◯	◯
keeping students on the right track and encouraging their engagement with the learning tasks	◯	◯	◯
meeting students face-to-face as a group to set the scene for the above tasks	◯	◯	◯

Comments are welcome.

Survey for Medical Graduates on Statistical Learning Needs (pdf copy of original online version)

11. Your experience as medical educator

*** 8. Please provide as accurate as possible an estimate of your start and end dates in your overall experience as an educator of University of Edinburgh undergraduate medical students. Ideally, you should provide the month and the year.**

Please select "I do not know the month." or "I do not know the year." if you cannot recall the month or year, respectively. If your above role is ongoing, you need only provide the start date.

	Month	Year
Start		
End		

Survey for Medical Graduates on Statistical Learning Needs (pdf copy of original online version)

12. Teaching of University of Edinburgh undergraduate medical students

The notion of Problem-Based learning (PBL) is used in this question. In PBL, "students working in a small group are presented with a problem, typically a description of a patient presentation. They decide what features of the problem are outside their present knowledge and divide these topics between them. They then research their topics using library and internet material and report back to the next small group tutorial with their findings."

(<ahref="http: www.biomedcentral.com="" 1472-6920="" 4="" 31"="" target="_blank"> http://www.biomedcentral.com/1472-6920/4/31)

*** 9. Please indicate your view on the potential utility of introducing PBL in Year 3 of the Edinburgh MBChB programme as a basis for integrating learning in statistics and probability with the learning of Evidence-Based Medicine.**

- ◯ not at all useful
- ◯ possibly useful
- ◯ moderately useful
- ◯ very useful
- ◯ essential
- ◯ don't know

Please feel free to add any comments by way of clarification.

Survey for Medical Graduates on Statistical Learning Needs (pdf copy of original online version)

13. Engagement with University of Edinburgh medical students or involvement in their learning

10. Please use the space below to convey any information you can on your engagement with University of Edinburgh medical students or your involvement in their learning.

Survey for Medical Graduates on Statistical Learning Needs (pdf copy of original online version)

14. Implementation of CAL materials

Many of you will be familiar with the CALs systems at the University of Edinburgh. CALs comprise sequentially arranged collections of Computer Assisted Learning objects and, among other things, serve as a potential vehicle for integrating real-life case-scenarios with statistical learning. If you require further clarification regarding the notion of CAL, please let me know (email: Margaret.MacDougall@ed.ac.uk).

*** 11. Would you be willing to provide feedback at a later stage on an existing version of CAL materials on**

	yes	no	maybe
confidence intervals	☐	☐	☐
estimating patient risk	☐	☐	☐

Survey for Medical Graduates on Statistical Learning Needs (pdf copy of original online version)

15. More about your work experience

*** 12. Please list as precisely and comprehensively as possible all of your existing clinical specialisms, separated by commas or semi-colons.**

Please avoid abbreviations. For each of the two boxes, please use "none" if this is the most accurate response.

Research [ ]

Clinical practice [ ]

*** 13. For *each* option which correctly categorizes your current job, please provide as accurate an estimate as possible of when you took up the corresponding responsibilities. Ideally, you should provide the month and the year.**

Please select "I do not know the month." or "I do not know the year." if you cannot recall the start month or year, respectively.

	Month	Year
Consultant clinician		
General Practitioner		
Registrar clinician		
Postdoctoral researcher		
Postgraduate student		
Foundation Year doctor		
Medical education researcher		
Clinical researcher		
Scientific researcher		
Other		

If you have selected 'Other' or you sense that your personal job categories are not adequately represented above, please specify them here, together with the corresponding start months and years, where possible.

Survey for Medical Graduates on Statistical Learning Needs (pdf copy of original online version)

16. A few demographic details

* 14. What is your gender?

- Male
- Female

* 15. Please select your age category.

	20 - 24	25 - 29	30 - 34	35 - 39	40 - 44	45 - 49	50 - 54	55 - 59	60+
Age category	○	○	○	○	○	○	○	○	○

Survey for Medical Graduates on Statistical Learning Needs (pdf copy of original online version)

17. Expression of thanks

Thank you very much for your time and patience in completing this questionnaire. Please click '**Done**' below to submit your responses.

N.B. The survey has been customized to ensure that after clicking the 'Done' button, you can return to your questionnaire at a later date via the link recommended in your invitation email. This allows you to add more information which you may have needed extra time to consider or to change your original responses, if appropriate.

This editing facility will remain in place until midnight on Monday 12 May 2014.

If, for any reason integral to the questionnaire design, you were unable to answer one or more questions to your own satisfaction, please use the email address (Margaret.MacDougall@ed.ac.uk) provided earlier in this questionnaire to specify your needs. It is important to ensure that no relevant point is missed!

I wish you much success with the remainder of your career.

Yours faithfully

Margaret MacDougall
Principal Investigator
