# Supplementary material for: Medical graduate views on statistical learning needs for clinical practice: a comprehensive survey
Source: BMC Med Educ. 2019 Dec 31;20:1. doi: 10.1186/s12909-019-1842-1 (PMC6937818; doi:10.1186/s12909-019-1842-1)
Supplement: Supplementary file 2 — Additional file 2. Further methodological details. Additional file 2 provides further methodological details concerning the procedures for data preparation, model building and model selection used for presentation and statistical analysis of the response data for this study. [file 12909_2019_1842_MOESM2_ESM.pdf]

Additional file 2: Appendix 2. Further methodological details

Further details of data preparation

The Principal Investigator (PI) downloaded the response data from the survey system in comma-separated values (CSV) format and anonymized these data in Microsoft Excel through removal of email addresses used by respondents, which served as the sole potential respondent identifier.

The thematic approach for classification of respondent clinical specialties (Q. 12) involved independent classification by two coders (the PI and the research assistant, who was a medical graduate). In cases of disagreement, arbitration was performed by a third coder who was selected in their capacity as a Specialty Registrar in Public Health Medicine. The specialties were finalized further by arbitration over 10 unclear cases, through the arbitrator offering recommendations based on their working understanding of clinical practice. Thus, for example, all three coders agreed that where General medicine was listed by the respondent as a specialty alongside more accurate classifications, such as Geriatrics or Acute medicine, it was superfluous to consider General medicine as a separate specialty. Likewise, similar terms used to identify the same specialty were subsumed under the same header. For example, ‘Primary healthcare’ and ‘General practice’ were subsumed under the single category ‘Primary healthcare’.

To ensure a reasonable group size for the youngest age group for mixed model analysis, we merged the original three age categories 25 - 29, 30 - 34 and 35 - 39 to form the single age category 25 - 39, noting that no respondents fell under the age category ‘20 - 24’.

Model building process and choice of omnibus test for fixed effects

We used generalized linear mixed models (GLMMs) primarily to assess the role of *statistical topic* (*TOPIC*) as an explanatory variable in choice of a response which falls under the category *includes practice* for the binary dependent variable with response categories *includes practice* and *does not include practice*, while also controlling for possible confounding arising from person characteristics. Variables for potential confounders included *number of statistical topics for which participant provided a response* (*numtopics*), *year of completion of study questionnaire (2013 or 2014)*, *age*,

gender, *nature of employment* (*EMPLOYME*) and *time as educator*. We used the variable for year of completion to test for evidence for bias arising from the year in which a respondent opted to complete the study. To account for differences between respondents, we also included a random intercept (*RESPID*) for participant identity. During the model building process, we also included the term *TOPIC*EMPLOYME* to test for an interaction effect between statistical topic and nature of employment.

For *age* and *time as educator*, 5.40% and 10.07% of data, respectively were missing, while for our binary response variable, 1.76% of data were missing. We used multiple imputation (MI) of missing data [1] to deal with this. We also used, Complete Case Analysis (CCA), however, to give an impression of the raw findings and the model fit, which is not straightforward to calculate with MI, and to test for evidence of an association between number of topics (*numtopics*) for which a participant offered a response and the choice of response category. This was under the recognition that repetition in response choices across topics by a respondent may be associated with reluctance to respond (e.g. low number of topics with response) or response fatigue (high number of topics with response), thus undermining the authenticity of the responses. Thus, we wished to explore the possibility of response patterns for listed topics arising from respondent apathy or fatigue.

In each case, we used the glmer function [2] of the R library lme4 to fit the models for the above binary dependent variable using maximum likelihood estimation with the Laplace approximation. We included fixed effects for all explanatory variables and a random intercept for the respondent identifier, *RESPID*. We used a GLMM with a logit link function for representing the non-Gaussian nature of the binary dependent variable. The model selection procedure involved an initial screening process whereby each candidate fixed effect variable was included separately in a model involving a random intercept for *RESPID* and assessed using a two-tailed t-test to confirm whether or not the corresponding model coefficient(s) were significantly different from zero. For the screening process, we assumed a statistical significance level of 0.10. We used this less stringent significance level to reduce the risk of wrongly ruling out important explanatory variables.

For CCA, we carried out model comparisons using the likelihood ratio test and through use of the Akaike and Bayesian Information Criteria (AIC and BIC, respectively). Using MI, we took a similar

approach to screening of variables for inclusion in our model and inter-model comparisons, although for model comparisons, we used Wald-style F-tests . At present, the use of AIC and BIC statistics for multilevel models in conjunction with MI is an open area of research and implementations in statistical software are not widely available. [1, 3]

The equation of the final fitted regression model expressing the relationship between the log-odds of a response which includes practice and our explanatory variables, was:

$$\eta_{ijk} = \log\left(\frac{\pi_{ijk}}{1-\pi_{ijk}}\right) = \beta_0 + \beta_{1,j} EMPLOYME_{ij} + \beta_{2,k} TOPIC_{ik} + u_i$$

$$(1 \le i \le 278; 1 \le j \le 6;\ 1 \le k \le 51), \qquad (1)$$

where π_{ijk} is the predicted probability of a response of the type *includes practice* for topic *k* by respondent *i* from employment category *j* and $EMPLOYME_{ij}$ and $TOPIC_{ik}$ are dummy variables corresponding to nature of employment category *j* and topic *k*, respectively for respondent *i*. For the *i*th respondent, *j* is fixed, while *k* ranges over all values between 1 and 51. Here, *Clinical Practice* is taken to be the reference category $(1 \le i \le 278; 1 \le j \le 6;\ 1 \le k \le 51)$. Further, the 51 categories denoted by categories 1 to 51 of *k* include all statistical topics listed in Tables 3a - e, with the exception of *Critical appraisal*, which is the reference category.

Also, u_i is the random effect for *RESPID*, β_0 is the fixed intercept, and $\beta_{1,j}$ and $\beta_{2,k}$ are the coefficients for the fixed effects corresponding to $EMPLOYME_{ij}$ and $TOPIC_{ik}$, respectively.

On assuming that the response data were missing at random (MAR) [1, 4], we carried out MI using the R package jomo [5, 6]. In order to satisfy convergence requirements, [7] we completed the MI process with 10000 burn-in iterations and 100 imputed data sets, each 500 iterations apart.

On implementing MI, we obtained odds ratios to represent the likelihood of choosing a response option which includes practice according to nature of employment and topic in statistics and probability. We also used the D1 procedure for combining multiparameter tests across multiple imputed datasets [1, 8] as an omnibus test for the overall effect for each fixed effect. For each of *EMPLOYME* and *TOPIC*, this involved testing the null hypothesis that the regression coefficients

were all equal to zero.

Model selection using CCA and MI

Variable screening

Based on the analyses using CCA, there was a lack of statistical evidence to suggest that the choice of whether or not to opt for a response which included practice was associated with *numtopics* (χ^2 =6.986, p = 0.7267). This helped in mitigating concerns about authenticity of responses which may, for example, have arisen from participant response fatigue or apathy. Using CCA, the following fixed effect variables proved insignificant as predictors of response status: *year of completion of study questionnaire* (χ^2 = 1.295, p = 0.255), *age* (χ^2 = 1.941, p = 0.857), *gender* (χ^2 = 1.250, p = 0.264) and *time as educator* (z = -0.392, p = 0.695). By contrast, the following fixed effects variables proved significant: *TOPIC* (χ^2 = 2336, p < 0.0005) and *EMPLOYME* (χ^2 = 22.397, p = 1.03 x 10^{-3}). These results were compatible with the corresponding results with MI (*year of completion of study questionnaire*: t = 1.158, p = 0.247; *age*: F = 0.380, p = 0.863, *gender*: t = -1.124, p = 0.261; *time as educator*: t = -0.333, p = 0.739, *TOPIC*: F = 45.834, p < 0.0005 and *EMPLOYME*: F = 3.777, p = 9.19 x 10^{-4}).

Diagnostic testing

Further to variable screening, we used CCA to explore a generalized linear mixed model with the same binary dependent variable but involving three main effects and one interaction effect. The main effects were represented by two fixed effect variables (*TOPIC* and *EMPLOYME*) and one random intercept variable (*RESPID*), where the interaction effect was between *EMPLOYME* and *TOPIC*. We compared this model with the corresponding model with no interaction. On use of model diagnostics, we found that the model arising from removal of the interaction term was justifiable in terms of model fit. In particular, in terms of the AIC statistic (increase in value from 12346 to 12392), this model yielded a modest decline in model fit, while the BIC statistic suggested a considerable improvement in terms of model fit (decrease in value from 13291 to 12838). Thus, in terms of model fit, the model without the interaction term appeared to have a slight edge over the

corresponding model with the interaction term included. This was reassuring when drawing comparisons with results involving MI, as with MI, it was necessary to remove the interaction effect between *EMPLOYME* and *TOPIC* to obtain a model which converged. For our final model, defined by equation (1), above, we found corresponding results using MI and CCA to be consistent, as would be expected where there are missing data only for the dependent variable. [9]

References for Appendix 2

1. Carpenter JR, Kenward MG: **Multiple imputation and its application**, 1st edn. Chichester, West Sussex, UK: John Wiley & Sons; 2013.
2. Bates D, Mächler M, Bolker B, Walker S: **Fitting Generalized Linear Mixed-Effects Models using lme4**. *Journal of Statistical Software* 2015, **67**(1): 1-48.
3. Grund S, Lüdtke O, Robitzsch A: **Multiple Imputation of Multilevel Missing Data: An Introduction to the R Package pan**. *SAGE Open* 2016:1-17.
4. Janssen KJ, Donders RT, Harrell Jr FE, Vergouwe Y, Chen Q, Grobbee DE, Moons KG: **Missing covariate data in medical research: To impute is better than to ignore**. *Journal of Clinical Epidemiology* 2010, **63**:721-727.
5. Lüdtke O, Robitzsch A, Grund S: **Multiple imputation of missing data in multilevel designs: A comparison of different strategies**. *Psychological Methods* 2016.
6. Quartagno M, Carpenter J: **jomo: A package for Multilevel Joint Modelling Multiple Imputation.** https://CRAN.R-project.org/package=jomo. Last accessed 18 May 2019.
7. Grund S: **Introduction**. In: *mitml vignettes*. The Comprehensive R Archive Network; 2017.
8. Grund S, Lüdtke O, Robitzsch A: **Pooling ANOVA results from multiply imputed datasets: A simulation study**. *Methodology* 2016, **12**:75-88.
9. Van Buuren S: Flexible imputation of missing data. Florida, USA: CRC Press; 2012.
