# Supplementary material for: Medical graduate views on statistical learning needs for clinical practice: a comprehensive survey
Source: BMC Med Educ. 2019 Dec 31;20:1. doi: 10.1186/s12909-019-1842-1 (PMC6937818; doi:10.1186/s12909-019-1842-1)
Supplement: Supplementary file 3 — Additional file 3: Table S1. Table S1 is a single table including the ranks, frequencies and percentages presented in Table 3 without sub-division of content by general topic. [file 12909_2019_1842_MOESM3_ESM.pdf]

Additional file 3: Appendix 3

Table S1. Relative frequency (as %) of Medical graduate responses on competencies in statistics and probability that medical schools need to provide

			Required competency					
Topic	n	rank	**Includes practice**	Practice only	Theory and practice	Theory only	Neither	Don't know
Graphical presentation of data:	274	1	**84.3**	32.8	51.6	14.2	1.1	0.4
Arranging data in spreadsheets for statistical analysis:	278	2	**81.7**	25.5	56.1	14.4	2.2	1.8
Understanding the proper meaning of an audit:	275	3	**81.5**	29.1	52.4	16.4	1.1	1.1
Sensitivity, specificity and positive and negative predictive values:	272	4	**75.0**	26.1	48.9	24.3	0	0.7
Simple descriptive (or summary) statistics:	272	5	**69.9**	24.6	45.2	21.7	5.5	2.9
Confidence intervals:	278	6	**65.1**	20.9	44.2	34.5	0.4	0.0
Using Excel for statistics - tips and warning:	270	7	**64.1**	26.3	37.8	23.0	9.3	3.7
Presenting the findings and conclusions of statistical hypothesis tests:	276	8	**63.4**	20.7	42.0	28.6	5.4	3.3
Conducting critical appraisals:	271	9	**62.0**	21.0	40.2	30.3	4.1	4.4
Statistical significance, statistical power and some facts about p-values:	275	10	**58.5**	18.9	39.6	39.6	0.7	1.1
Getting to know the fundamentals of a statistical package such as SPSS:	274	11	**57.3**	27.0	30.4	22.6	14.6	5.5
Laws of probability:	278	12	**48.9**	12.2	36.7	46.	43.2	1.4
Designing survey questions to support valid statistical analyses:	271	13	**48.0**	15.9	32.1	36.2	10.0	5.9
Valid reporting and interpretation of statistical findings:	274	14	**46.0**	12.4	33.5	48.4	3.6	2.2

Statistical effect sizes:	274	15	**44.9**	11.3	33.6	51.5	2.2	1.5
Principles of good study design:	275	16	**42.6**	14.2	28.4	55.3	1.8	0.4
Tests of normality:	274	17	**41.7**	11.3	29.9	43.8	8.0	6.9
Sample size calculations:	272	18	**41.2**	14.0	27.2	47.1	8.5	3.3
Simple linear regression analysis:	278	19	**39.6**	14.4	25.2	43.9	11.2	5.4
Randomization:	275	20	**39.6**	13.1	26.5	59.3	0.7	0.4
Misuse of statistics: some statistical blunders and phenomena to look out for in published literature:	274	21	**39.4**	10.6	28.8	52.9	5.1	2.6
Comparing a study cohort with a general population:	274	22	**38.3**	13.5	24.8	56.6	4.4	0.7
Cross tabulating frequencies or percentages:	273	23	**37.4**	15.0	22.3	28.9	16.1	17.6
Hypothesis tests for a single group of continuous data:	269	24	**35.3**	10.4	24.9	35.4	13.0	16.4
Concepts and rules of probability:	276	25	**34.5**	10.2	24.4	61.1	2.9	1.5
Different types of study design:	275	26	**34.2**	9.1	25.1	64.7	0.4	0.7
Working with subsets of the original data – filtering data:	274	27	**33.2**	13.9	19.3	38.0	15.3	13.5
One-tailed versus two-tailed hypotheses tests:	271	28	**32.8**	9.6	23.2	48.7	9.2	9.2
Health-related data sources:	272	29	**32.7**	9.9	22.8	45.2	7.7	14.3
Hypothesis tests for categorical data:	271	30	**32.1**	8.5	23.6	35.8	14.0	18.1
Systematic reviews:	267	31	**31.5**	8.2	23.2	65.5	2.2	0.7
Statistical aspects of clinical trials:	272	32	**29.4**	6.3	23.2	65.8	2.9	1.8
Hypothesis tests for comparing two groups of measurement or ordinal data:	270	33	**29.3**	5.9	23.0	31.5	17.4	21.9

Correlation coefficients – linear and non-linear:	271	34	**29.2**	8.1	21.0	53.1	10.0	7.7
Representing socioeconomic status:	278	35	**29.1**	6.5	22.7	58.6	6.1	6.1
Analysis of variance (ANOVA):	270	36	**27.0**	8.5	18.5	45.6	15.2	12.2
Statistical indices for measuring levels of agreement and consistency:	274	37	**27.0**	7.3	19.7	55.1	9.9	8.0
Statistical risk estimates:	272	38	**23.0**	5.2	17.8	62.6	6.3	8.1
Survival analysis:	277	39	**20.9**	5.4	15.5	65.0	7.2	6.9
Assessing agreement between two methods of measurement:	268	40	**18.7**	5.6	13.1	48.9	16.4	16.0
Multiple linear regression analysis:	278	41	**18.3**	6.8	11.5	58.3	16.2	7.2
Merging similar datasets:	273	42	**17.9**	6.2	11.7	38.8	27.5	15.8
Summarising and analysing missing data:	267	43	**17.6**	4.9	12.7	50.2	20.6	11.6
Analysis of covariance (ANCOVA):	269	44	**16.7**	5.9	10.8	42.4	23.0	17.8
Forest plots:	274	45	**15.0**	3.6	11.3	51.8	17.5	15.7
Receiver operating characteristic (ROC) curves:	275	46	**13.6**	2.6	11.0	45.4	18.3	22.7
Retrospective power calculations versus examination of confidence intervals:	275	47	**12.7**	2.5	10.2	48.7	21.5	17.1
Cross-over trials:	271	48	**11.8**	2.6	9.2	70.5	10.3	7.4
Types of response data:	272	49	**9.6**	2.9	6.6	41.5	17.3	31.6
Tests of homoscedasticity (or, 'equality' of variance):	269	50	**6.7**	1.5	5.2	30.5	30.1	32.7
Cluster analysis:	277	51	**6.1**	1.1	5.1	52.7	26.7	14.4
Time series analysis:	272	52	**5.9**	2.2	3.7	36.4	27.6	30.1

Note. The column header 'n' denotes the number of responses for the given topic, while 'Practice only', 'Theory and practice' and 'Theory only' are abbreviations used for the listed response options 'carry out the procedure or calculate the statistic(s) using appropriate data', 'both of the above' and 'understand the theory only', respectively. Correspondingly, column 4 is formed by combining columns 5 and 6. Percentages are row percentages with the denominator in the calculation pertaining to the number of persons who responded for the listed topic, inclusive of those who responded, 'Don't know'.
