# Supplementary material for: Medical graduate views on statistical learning needs for clinical practice: a comprehensive survey
Source: BMC Med Educ. 2019 Dec 31;20:1. doi: 10.1186/s12909-019-1842-1 (PMC6937818; doi:10.1186/s12909-019-1842-1)
Supplement: Supplementary file 4 — Additional file 4. Further topics in statistics and probability emergent from free text response data and the need for boundaries. Additional file 4 provides: a) a brief narrative synthesis of the free text comments arising from the second part of Q. 2 of the study questionnaire, pertaining to what was missing from the available list of topics and b) recommendations for approaches to teaching statistics suggested by the emergent themes associated with these free text responses. [file 12909_2019_1842_MOESM4_ESM.pdf]

Additional file: Appendix 4. Further topics in statistics and probability emergent from free text response data and the need for boundaries

Free text responses pertaining to what was missing from the list of available topics in statistics and probability (Q. 2) were prepared in a spreadsheet by a research assistant and examined by the Principal Investigator (PI) to identify volunteered topics which did not fall under or coincide with already listed topics. Where new topics were evident, we preserved the actual wording of the respondent.

In relation to the query inviting free text responses (second part of Q. 2) on what was missing from the available list of topics (first part of Q. 2), respondents frequently offered suggestions which were already encompassed in the comparatively broader listed topics. Below, we list in respondents' own terms topics they suggested that were not covered under those we had previously presented to them. Frequencies of respondents (listed in brackets alongside each item) are too low to merit use of thematic analysis.

- "Concepts of accuracy (how many significant figures do you need)". (1)
- "[E]xploring data to look for errors, outliers, how to deal with outliers". (1)
- "[S]ome understanding of logistic regression, use of bootstrapping". (1)
- "Single case analysis". (1)
- "Something about health economic [analyses] such as QALYs", where we understand 'QALY' to denote 'quality-adjusted life-year'. (1)
- Bayesian statistics (2)
    - for "Validating the value of diagnostic tests" (1)
    - restriction to "theoretical concepts" pertaining to Bayesian methods. (1)
- Statistical control charts. (2)
- Where to draw the line in terms of depending on Excel rather than progressing to a statistical package, such as "R". (1)

  and

- Knowing ‘[h]ow to deal with a “real-world” d[a]taset’. (1)

Six respondents also expressed the need for boundaries to be set in terms of expectations of medical graduates in their personal use of statistics for clinical practice. These boundaries required appreciation of knowledge limits and how to engage in inter-professional collaboration with a medical statistician effectively, including in relation to early identification of the statistician in preparation for study design. A respondent also suggested that prior training in application of statistics within clinical contexts could support medical graduate awareness of the importance of the above need for preparedness in advance of data collection.

In addition to these expressed boundaries, one candidate identified omission of the practice of statistics as a barrier to understanding the theory as follows:

> I have reluctantly ticked 'understand theory only' boxes quite often, because students may not need to actually perform some of these operations when they become doctors, and there is a risk that when one is overloaded with statistical information during the medical curriculum (as I was) then it is all speedily forgotten. On the other hand, understanding theory is often only fully possible when undertaking the calculation ... (Female: 60+; adult psychiatry and child and adolescent psychiatry, Clinical Practice, Academic Teaching & Academic Research).

Communicating statistics

The needs were recognized for:

a) more teaching on translating risk to patients or “lay persons” (3)

   and

b) addressing a general deficit in understanding how to communicate the results of survival analysis and translate group-based statistical findings into results of relevance to the individual patient. (1)

Further, a respondent suggested the idea of introducing “something specifically geared at interpreting inaccuracies in media reporting of statistics in relation to medicine, with examples”. This suggestion

is clearly of direct relevance to the topic *Misuse of statistics: some statistical blunders and phenomena to look out for in published literature* and demonstrates how the delivery of this topic could be linked with enhancing the skill of understanding better communication of statistics.

It is of particular interest to note that the themes of having a better understanding of risk, and being better able to explain things to, or teach, other people, volunteered by respondents in the 2007 study [1, 2], resonate with free text responses from the current study. It is also instructive to note from the current study that respondents did not necessarily view explaining risk to patients as inclusive of competency in calculating the corresponding risk statistics. This is evident from linking the selections for the listed items for Q.2 with the corresponding free text responses for this question. Two respondents who recommended for the topic *statistical risk estimates* that students should understand the theory only also placed an emphasis on their own need for improved skills in explaining risk to patients. By contrast, two respondents who had expressed the need for similar skills in their free text responses had identified both understanding the theory and calculating the corresponding risk statistics as important competencies for the same topic.

Regarding communicating statistics more generally, one respondent's perception of a possible evolutionary trend in need for training undergraduate medical students to communicate statistics was reflected in the following remarks:

> Medical statistics was an important topic when I was a medical student and I think it is becoming more important. I feel this because of the internet usage amoungst [sic] our patients and often the "knowledge" they come with to the consultation. The profession has to have a better understanding of the evidence and how best to interpret the data and tailor it to the individual patient that is in front of us. I wonder if a lack of knowledge in this area will become the complaints of the future - and in a sense a communication break down [sic], because a failure to be able to speak the language of statistical analysis is just that (especially when you cannot correctly convey the information to the patient). (Female, 40 – 44, Primary healthcare, Clinical practice and Academic teaching)

Making conceptual distinctions

The capacity to make conceptual distinctions in statistics also emerged as a theme from the free text responses. This resonates with variation theory as a branch of learning theory, according to which "without variation there can be no discernment of a particular phenomenon (the object of learning)." [3] Similarly, Star et al. [4] note that "Comparison is a powerful tool that has been shown to improve student learning in a variety of domains." In the response data for the current study, an emphasis was placed on being able to make conceptual distinctions between:

- parametric and non-parametric tests or data, including in the context of study design; (1)
- right and wrong statistical tests for a study, rather than simply knowing the tests per se; (1)
- the mean and median; (1)
- types of data (continuous versus categorical ("category")); (1)
- statistical and clinical significance; (3)
- "correlation and causation"; (1)
- approaches to analysis for randomized trials (intention-to-treat versus per-protocol); (1) and
- statistical risk estimates (absolute versus relative risk or absolute versus relative risk reduction). (2)

References for Appendix 4

1. Miles S, Price GM, Swift L, Shepstone L, Leinster SJ: **Statistics teaching in medical school: Opinions of practicing doctors**. *BMC Medical Education* 2010, **10**(75).
2. Swift L, Miles S, Price GM, Shepstone L, Leinster SJ: **Do doctors need statistics? Doctors' use of an atittudes to probability and statistics**. *Statistics in Medicine* 2009, **28**:1969-1981.
3. Meyer JHF, Land R, Davies R: **Threshold concepts and troublesome knowledge (4)**. In: *Threshold Concepts within the Disciplines.* edn. Edited by Land R, Meyer JH, Smith J. Rotterdam: Sense Publishers; 2008: 59-74.
4. Star JR, Pollack C, Durkin K, Rittle-Johnson B, Lynch K, Newton K, Gogolen C: **Learning from comparison in algebra**. *Contemporary Educational Psychology* 2015, **40**:41-54.
